# A Blockchain-Based Multi-Factor Authentication Model for a Cloud-Enabled Internet of Vehicles

**DOI:** 10.3390/s21186018

**Published:** 2021-09-08

**Authors:** Victor R. Kebande, Feras M. Awaysheh, Richard A. Ikuesan, Sadi A. Alawadi, Mohammad Dahman Alshehri

**Affiliations:** 1 Department of Computer Science, Electrical & Space Engineering, Luleå University of Technology, 97187 Luleå, Sweden; victor.kebande@ltu.se; 2 Department of Computer Science (DIDA), Blekinge Institute of Technology, 37179 Karlskrona, Sweden; 3 Institute of Computer Science, Data Systems Research Group, Tartu University, 51009 Tartu, Estonia; 4 Cyber and Network Security Department, Community College Qatar, Doha 00974, Qatar; richard.ikuesan@ccq.edu.qa; 5 Department of Information Technology, Uppsala University, 75236 Uppsala, Sweden; sadi.alawadi@it.uu.se; 6 Department of Computer Science, College of Computers and Information Technology, Taif University, P.O. Box 11099, Taif 21944, Saudi Arabia; alshehri@tu.edu.sa

**Keywords:** blockchain, multi-factor authentication, access control, Internet of Vehicles, cloud-enabled systems

## Abstract

Continuous and emerging advances in Information and Communication Technology (ICT) have enabled Internet-of-Things (IoT)-to-Cloud applications to be induced by data pipelines and Edge Intelligence-based architectures. Advanced vehicular networks greatly benefit from these architectures due to the implicit functionalities that are focused on realizing the Internet of Vehicle (IoV) vision. However, IoV is susceptible to attacks, where adversaries can easily exploit existing vulnerabilities. Several attacks may succeed due to inadequate or ineffective authentication techniques. Hence, there is a timely need for hardening the authentication process through cutting-edge access control mechanisms. This paper proposes a Blockchain-based Multi-Factor authentication model that uses an embedded Digital Signature (MFBC_eDS) for vehicular clouds and Cloud-enabled IoV. Our proposed MFBC_eDS model consists of a scheme that integrates the Security Assertion Mark-up Language (SAML) to the Single Sign-On (SSO) capabilities for a connected edge to cloud ecosystem. MFBC_eDS draws an essential comparison with the baseline authentication scheme suggested by Karla and Sood. Based on the foundations of Karla and Sood’s scheme, an embedded Probabilistic Polynomial-Time Algorithm (ePPTA) and an additional Hash function for the $P_{i}$ generated during Karla and Sood’s authentication were proposed and discussed. The preliminary analysis of the proposition shows that the approach is more suitable to counter major adversarial attacks in an IoV-centered environment based on the Dolev–Yao adversarial model while satisfying aspects of the Confidentiality, Integrity, and Availability (CIA) triad.

## 1. Introduction

Blockchain technology establishes a creditworthy ecosystem among independent participants within a non-trustable distributed environment according to Li [1]. For example, in the cybersecurity world, blockchain technology has very distinctive use-cases driven by the fact that many of the security parameters used for identification, authentication, and authorization in organizations have become progressively penetrable. With the introduction of different cloud-based applications, Bring Your Own Device (BYOD) [2], as well as other cloud technologies authentication challenges, continue to introduce several threat vectors to many organizations. Furthermore, the answers on dealing with identity management, authentication, and access-control security in the many heterogeneous environments constitute diverse challenges to many industries. Things-enabled communications, such as Internet of Things (IoT) and Internet of Vehicles (IoV), for instance, are particularly affected by this authentication challenge. However, with the IoT becoming increasingly crucial to intelligent transportation system stakeholders, including cloud-based vehicular (VC) and IoV paradigms, greater threat vectors are continually introduced. This new trend involves communication and data exchange between several objects within different layers of control in centralized [3] and decentralized models [4]. Security, particularly the authentication mechanism, in such a deployment, it is pivotal to realize the general IoT vision. Exploring the potentials of blockchain technology applications was a subject of intensive discussion in the literature. Many researchers investigate its ascribed advantages beyond the premises of cryptocurrencies. Among these possible applications, Blockchain-driven access control has distinguished itself as a promising trend [5,6].

Due to the dynamic nature of access control, agility has become unavoidable in many domains, including the connected vehicles [7]. For this, several studies have proposed access control technologies to address the broader intelligent transportation systems [8] due to ease of use and simplicity with an adequate security level [9]. Access control mechanism such as single sign on (SSO), Multi-Factor Authentication (MFA) process, Open Authentication (OAuth), open ID connect, as well other forms of authentication are key candidates in this context. With SSO, however, an entity can be authenticated using one set of login credentials and given access rights to multiple applications and services in a cloud platform to eliminate further prompts when the user switches applications or services during the same session. However, different organizations have opted to enforce MFA to verify a user’s identity, requiring multiple identity and access management credentials. MFA can, therefore, be considered as a practical approach to security enhancement. Such models, however, required both security evaluation and risk assessment [10,11], as well as scalable security management frameworks [12].

Moreover, SSO and MFA have been implemented individually and not integrated to form Standard Operating Procedures (SOPs) in organizations. By leveraging the security strength of SSO and MFA combined, a viable alternative to entity authentication in things-enabled communication can be achieved, while minimizing the compromising limitation of each authentication mechanisms. The one good thing with SSO is that it can log user activities and monitor user accounts. The introduction of MFA in organizations, on the other hand, has been considered as one of the effective control measures that an organization can put in place to prevent attackers from gaining access to critical infrastructure as well as networks, thus preventing access to sensitive information. Accordingly, if a criminal manages to steal a user credential, he/she will be foiled by having to verify his identity differently. Hence, making it significantly hard for any adversary to steal legitimate user credentials for malicious activities on any organization network [13]. Besides SSO and MFA, security by design is a critical factor in the fortification of the system [14].

To further strengthen the security mechanisms and keep prevent attackers from malicious and unauthorized access, this study discusses a lightweight blockchain-based multi-factor authentication scheme for smart cities that integrates SSO and SAML in the cloud. This was motivated by the knowledge that IoT-based smart cities usually implement a complex distributed system that may involve multiple stakeholders, applications, sensors, as well as other IoT devices [15], hence the need for an integrated authentication mechanism. In addition to the aforementioned, this manuscript further extends the earlier work presented in [16].

### 1.1. Key Security Issues in IoV

The interaction between diverse applications and services across the vehicle cloud face a number of challenges. Among these challenges is the heterogeneity and the need to achieve inter-operable solutions. That notwithstanding, attackers can easily exploit vulnerabilities emanating from identity verification and device authentication in IoV. In order to enforce secure communication in a cloud-enabled IoV environment, the following are considered as key issues:

- Illegitimate identities where it is imperative to conduct a verification of key identities during authentication.
- Unauthorized access where it is important to verify the authenticity of a use accessing the cloud server or IoT device.

### 1.2. Contributions

Whilst several studies on authentication for IoT-based smart environments have leveraged the principle of MFA, the ultimate objective of any security mechanism is to guarantee secure communication by preventing compromise and attacks on the existing authentication mechanisms. Based on these factors, a secure MFA scheme for IoV ecosystems has been suggested. Therefore, the contributions of this paper are summarized as follows:

- The paper proposes a Multi-Factor Blockchain-based authentication model that uses an embedded Digital Signature (MFBC_eDS) for vehicular clouds and Cloud-enabled IoV.
- The suggested MFA Scheme combines and integrates a number of aspects in order to harden key authentication techniques. For example, SSO and SAML are key aspects that have been used to enhance authentication of IoT systems in the cloud. The security strength of the proposed approach shows that it satisfies the principle of data confidentiality and integrity, two cardinal components of the security of IoV.
- An embedded probabilistic polynomial Time Algorithm (ePPTA) with an additional hash function has been suggested that not only compliments the existing schemes but also hardens based on existing weaknesses, while it is applicable in an IoV-based environment.
- This study concentrates on addressing the degree of resistant-precisely on the possible failure of the mutual authentication phase, once $P_{i}$ is generated.

### 1.3. Organization

The remainder of this paper is organized as follows: Section 2 presents the required background and motivation concepts behind this work. Section 3 presents existing state-of-the-art publications on related areas that we discuss in this manuscript. Section 4 exhibits the methodology used and the approach used that relies on the Karla and Sood authentication scheme and discusses its primaries. We introduce the proposed model in Section 5, alongside the validation process. A comprehensive discussion on this study’s main findings took place and was discussed in Section 6. Finally, the study drafts its conclusions and future work in Section 7.

## 2. Background

This section explains the basic concepts and definitions of authentication models, single-sign-on frameworks, vehicular clouds, and the IoV paradigm.

### 2.1. Multi-Factor Authentication

A Multi-Factor Authentication (MFA) scheme offers solutions to the security risks and vulnerabilities found in a single-factor authentication mechanism. MFA thus offers security enhancement that allows a user to present two or more pieces of authentication credential when logging in to any account. This can range from something you know (password or PIN), something you have (smart card), or something you are (fingerprint) [17,18]. However, the latest MFA solutions incorporate additional factors which can consider context and behavior when authenticating a user—for instance, the location when logging in, attempted log-in time (such as late at night, for instance), the device being used (either a smartphone or a laptop), as well as the network being used to access (either private, public, or designated IP address range). With MFA, a complementary layer of security is added to strengthen the security against an attack [18]. A more robust (not necessarily complex) authentication often poses a usability problem [19]. Therefore, there is the need to evaluate the usability of a security mechanism constantly. As a simple thumb rule, usability is inversely proportional to security. It is, therefore, essential to note that there is a trade-off between usability and security when it comes to deciding on authentication schemes. The username and password authentication process is the most popular means, despite their security flaws because they are easy to implement and allows the user quick entry to the system. They can be implemented with less computational complexity, speed, and scalability [17]. Additional devices are required to implement an MFA, which could be expensive, and more computational complexity will be required, which also increases processing time.

### 2.2. Single-Sign On

**Figure 1:**
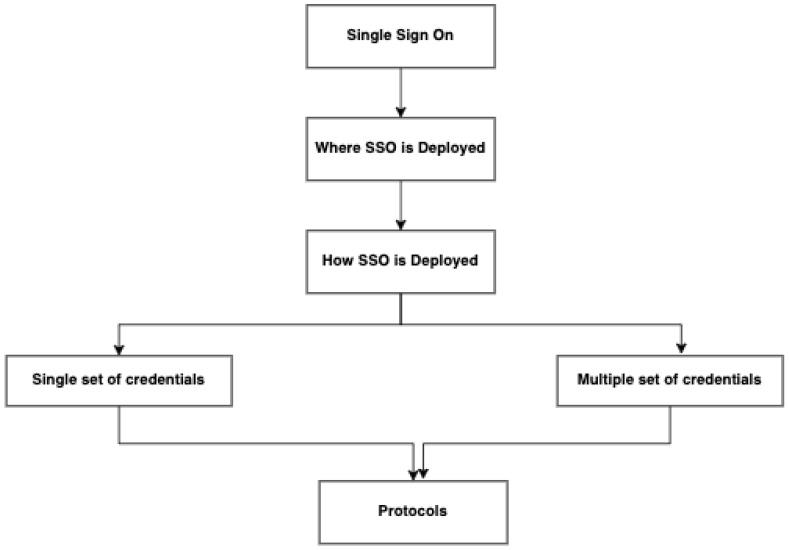
How SSO strategy is classified, where and how it is deployed.

With the availability of cloud computing platforms, users are now able to access multiple, heterogeneous systems, either on the Internet, Extranet, or Intranet [20]. However, access to multiple systems may also mean multiple login credentials that users need to possess. This process can add extra pressure on the user to create and remember multiple login credentials, usually in the form of usernames and passwords, as different systems (may) have different constraints [21,22]. Therefore, SSO addresses the problem of multiple login credentials for multiple systems [23]. It is an authentication scheme through which a server authenticates a user with a single set of login credentials to gain access to all or multiple system resources and services without being prompted for a repeated login process. The main benefit of the SSO is the provision of improved security and compliance. Figure 1 shows a simple classification of SSO depicting where and how it is deployed, the set of credentials, and protocols.

In order to improve the security and usability of a system, SSO is usually deployed both within the Intranet, Extranet, and at the Internet level. However, a wide range of security vulnerabilities with the SSO approach exists [22]. For example, OpenID is a key technology that has been used by many Internet Service Providers (ISPs) as an authentication scheme for SSO [22]. To implement OpenID, one must integrate it with Secure Socket Layer (SSL) connections to leverage the RSA public-key cryptography of an SSL. The problem that comes with this measure is that there are high computational costs involved when cryptography is used [22], hence the need to refine and secure the SSO process while minimizing the computational costs. The use of SSO has led to information security vulnerabilities such as identity deception, identity theft, and authentication issues, especially in the cloud platforms, which mostly have seen a rise in Man in the Middle (MiTM) attacks or dictionary attacks. An SSO model for big data federation architectures was reported as well in [24] to depend on the reference model and digital evidence.

### 2.3. Vehicular Cloud

Vehicular Cloud (VC) refers to a group of broadly autonomous vehicles whose corporate computing, sensing, communication, and physical resources can be coordinated and dynamically allocated to share internet access, as well as data, with other devices both inside and outside the vehicle. The VC can be formed by vehicles autonomously and provides a vast number of applications and services that can benefit the entire transportation system and its stakeholders (drivers, passengers, and pedestrians). This process, however, involves the use of onboard computational resources to facilitate communication and decode message and information storage. This concept of utilizing excessive onboard resources in the transportation system and the latest computing resource management technology in conventional clouds provides the substratum for the development of the VC. In general, it is composed of (i) Vehicular Ad-hoc Networks (VANET), where communication can be between vehicles (V2V) or vehicle and roadside infrastructure (V2I). (ii) Connected vehicles that interact with each other (V2V), the roadside infrastructure (V2I), and beyond (V2X) via wireless communications. (iii) VC is an attractive technology, which takes advantage of big data analytics [25] and cloud computing to support many novel applications. Like any other VANET, data privacy, entity authentication, and resource management are major challenges. Entity authentication and data privacy in such context are top priorities, maintaining its provenance [26].

## 3. Related Work

As an important method of hardening security, there has been a vast contribution in different authentication techniques in research that have provided essential solutions. An optimization approach focused on IoT security has been enforced with cryptographic encryption techniques for medical images using grasshopper and Particle Swarm Optimization (PSO). It depicted a diverse encryption algorithm for the secure transmission of medical images in an IoT environment [27]. Next, a lightweight authentication scheme has opted to focus on a multi-gateway for Wireless Sensor Networks (WSNs) in IoT—the proof of analysis of this scheme shows it prevents usual attacks [28,29]. Given that most IoT authenticating techniques use single-factor techniques, research by [30] has proposed a lightweight MFA for IoT devices that configures physical functions within IoT devices, and it makes use of very few cryptographic processes while employing a one-way hash. Another novel proposition protocol uses MFA for passwords, smart-cards, and bio-metrics for healthcare applications where there is a mutual authentication for each remote medical professional and the server [31].

Moreover, the authors in [32] have proposed an authentication scheme that focuses on cloud-IoT applications that are robust and lightweight. One advantage of this scheme is that it is robust against attacks with low computation overhead. Studies by Zisang et al. [33] have proposed a blockchain-based authentication approach for the Internet of Vehicles (IoV) that also manages key agreement protocols. In addition, in that study, blockchain is mainly used as a Trusted Authority (TA) that allows the management of the ledger that can store information related to the vehicle. It is also essential for the vehicles to perform mutual authentication with the TA through the intermediate node. However, the study pinpoints low computing overhead [33]. Another comparative study aimed at checking if blockchain technology can be used to improve the security, privacy, and trust of vehicle technology shows that blockchain could easily facilitate resource sharing among vehicles with a focus on computational, storage, and communication [34]. In addition, the study by [35] suggests an approach for solving security issues in IoVs for purposes of intelligent transport. Their study has a focus on communication, consensus-making, and authentication using a Byzantine consensus-based algorithm. From their study, the Byzantine outperforms the traditional authentication methods for IoV. Notably, that study mainly offers a key reference solution for authentication issues to the blockchain. While the ultimate benefits are decentralization, scalability, and fault-tolerance, it hardly has a focus on being integrated with multi-factor modalities [35].

Other relevant research includes an authentication scheme for IoV using blockchain that uses a blockchain ledger to design new nodes joining the consensus for vehicle identity. That authentication—which in the long run curbs malicious attacks [36]—is a blockchain-based batch authentication that supports AI for IoV deployment—where, at the signing phase, the vehicle can broadcast messages to the Road Side Unit (RSU) using Vehicle to Vehicle (V2V) and batch authentication. The outcome is effective communication, storage, and computation cost and time [37]; in addition, an efficient blockchain authentication scheme that has a focus on fog computing for IoV named EASBF with five main phases: initializing, registering, mutual authentication, key exchange, consensus, and certificate update. EASBF uses elliptic curve cryptography and one-way as opposed to ePPTA being employed in this paper [38]. Lastly, blockchain-based lightweight for secured V2V uses blockchain and achieves data authentication among vehicles in real time for purposes of vehicle real-time adversary detection [39].

## 4. Methodology

**Figure 2:**
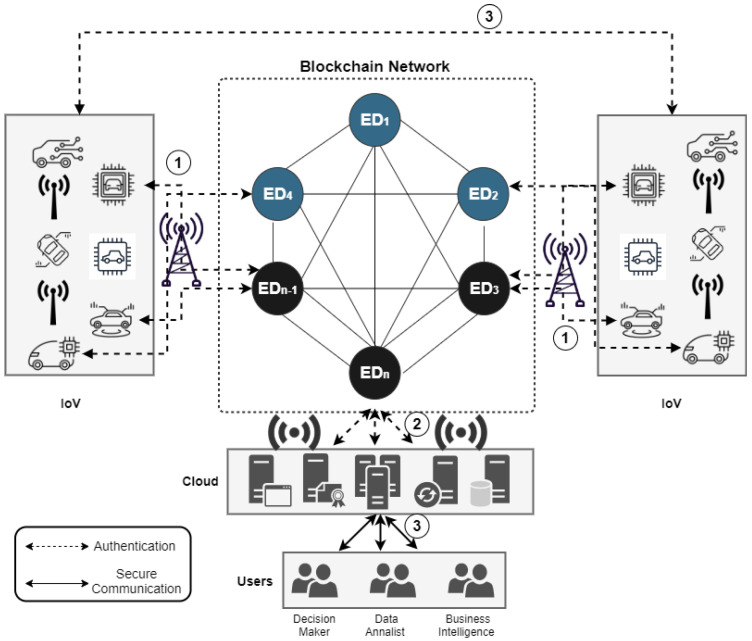
Blockchain-based Multi-Factor Authentication with ePPTA for IoV.

We mainly focus on the authentication of secure communication between vehicle-to-vehicle (V2V) and vehicle-to-Cloud (V2C) as shown in Figure 2. The model comprises the following components:

- a set of connected smart vehicles;
- a peer-to-peer (blockchain-based topology) and IoT-to-Cloud network connected by multiple cloud service providers;
- a public Cloud infrastructure.

The connected vehicles collect sensor data using a solid-state programmable device, like real-time electricity load, temperature, proximity and humidity sensors, electricity consumption, …, etc. In our model, the connected vehicles send the ID of the corresponding cloud service providers to confirm their manager. Hence, at the data aggregator layer, the cloud service provider is responsible for several connected vehicles and maintaining the data-flows among the V2V and V2C in real-time. The proposed architecture in Figure 2 is further discussed, thus:

1. Initial Registration: when a vehicle joins the network and first participates in the system, it is asked to generate a hash-chain for the initial registration.
2. Update the hash-chain Information: using one-time passwords, the vehicle periodically changes their hash-chains, so they need to contact the service provider to generate a new chain to establish a communication with the cloud.
3. Communication establishes: a secure data channel is established (authenticated), V2V and V2C take place.

### Approaches Based on Karla and Sood’s Scheme

The Elliptic Curve Cryptography (ECC) in Karla and Sood [40] is based on the authentication protocols for the HTTP client that targets embedded devices. This protocol acts as a client, it is configured over TCP/IP, and it operates over a client/server communication with three distinct phases as is shown in the workflow protocol as follows: an embedded device $\left(D_{i}\right)$ that wants to connect to the server (*S*) must register with the server (S), by first sending an identity, $ID_{i}$ to *S*. Then, *S* will generate a key, $P_{i}$ to be used coupled with a randomized number, and $R_{i}$ that is to be used with the identity of the embedded device, $D_{i}$. This approach is computed as follows:

- Registration: $D_{i}$ submits $ID_{i}$
  $$T_{i}=H(R_{i}⊕H\left(X\right))\;A^{\prime }i=A_{j}xG,T_{i},ID_{i}\;Ai=H(Ri⊕H\left(X\right)⊕Pi⊕CK^{\prime })\;CK=H(R_{i}\|X\|\mathrm{EXP}-\mathrm{Time}\|ID_{i})\;Server\;Stores\;A^{\prime }i=A_{j}xG,T_{i},ID_{i}\;Server\;generates\;P_{i}\;x⟶\;S^{\prime }private\_key\;EXP-Time\;\;\;\;\;\;\;\;\;\;\;\;S⟶\;\mathrm{sends}\;CK^{\prime }toD_{i}$$
- Pre-Computation Time Phase
  Once $D_{i}$ obtains the authenticating key CK${}^{\prime }$, it becomes paramount that this can be used in the message computation that is required to be authenticated. A random number $N_{i}$ is selected, which uses the authentication key CK${}^{\prime }$ for computation as follows:
  $$\mathrm{Select}\;N_{1}\;P_{1}=N_{1}xG\;P_{2}=H\left(N_{1}xCK^{\prime }\right)\;D_{i}\;sends\;required\left(\mathrm{Auth}\right)message\left(ID_{i},P_{1},P_{2}\right)$$
- Authentication Phase
  After the server, $S⟵(ID_{i},P_{1},P_{2})$, it proceeds to such using $ID_{i}$, and it can find the desired record using the private key and expiration time EXP−Time and the computation is as follows:
  $$R_{i}=T_{i}⊕H\left(X\right)\;CK=H(R_{i}|X|EXP-Time|ID_{i})\;P_{2}^{\prime }=H\left(P_{1}xCK\right)\;S\;checks\;if\;P_{2}^{\prime }=P_{2}\;Random\_number\;N_{2}\;is\;selected\;Calculates\;ECC\;based\;on\;P_{3}=N_{2}\times G\;\mathrm{and}\;P_{4}=N_{2}\times A^{\prime }$$

The embedded device will then calculate values of A${}^{\prime }$, checks if $P_{4}^{\prime }=P_{4}$ and then it sends a message to the server, S. Once the server checks if $V_{i}^{\prime }=V_{i}$ a mutual authentication between $D_{i}$, a cloud server is generated and both parties agree on a common session key.

According to Karla and Sood’s scheme [40], an attacker may only try to find intrusion mechanisms through unauthorized access and specifically by accessing the cloud server instead of an IoT device or an embedded device. As a result, Karla and Sood’s [40] scheme will resist a replay attack, a man in the middle attack, eavesdropping, cookie theft, brute force attack, dictionary attack, verifier attack and mutual authentication, confidentiality, and anonymity. Based on Karla and Sood’s work, we are more concerned with the degree of resistance if such a scheme is to be employed in a smart city and, as a result, the authors of this paper are more concerned with step 3 (mutual authentication phase) on possible failure once $P_{i}$ is generated.

## 5. Proposed Lightweight MFA Scheme

This section presents the proposed Lightweight MFA Scheme by mainly exploring the adversary model and the lightweight blockchain-based Multi-Factor Authentication (MFA) scheme that integrates SSO and SAML in the cloud. We have formally defined BCMF_eDS supported access control model in Table 1 that shows the model’s primary assets and functions. In addition, it describes the effective authentication scheme using the MFA and ePPTA foundations. A demonstration of the authentication and the associated decision process is presented in four steps. As shown in Table 1, IoV service permissions are the power set of the cross-products of the proposed algorithm and adapted approach. It worth mentioning that the system capitalizes on Phase-3, on the possible failure of the Karla and Sood [40] mutual authentication phase. In addition, this study also looks at the constitutes of the adversary model.

### 5.1. Assumptions Based on the Dolev–Yao Adversary Model

The proposed adversary model is based on the Dolev–Yao [41] framework that is used in the analysis of security protocols. The adversary model is aimed at showing failures of the security goals: Confidentiality, Integrity, and Authentication (CIA), by relying on the assumptions that the adversary has a reason for the attack, what an adversary aims to achieve, as well as the capabilities of an adversary. Based on the Dolev–Yao adversary model, this study extrapolated the following assumptions [42,43]:

- Confidential or secret information being transmitted can be obtained through a passive attack process such as eavesdropping.
- An adversary can easily interfere with communication between two parties in a connected smart city or IoT environment.
- Sensor nodes can be interfered with or compromised in a bid to extract sensor data which can further compromise the confidentiality.
- Modification/tampering of digital information, a process which can compromise the integrity, potentially, and the availability of the data.

### 5.2. MFA Scheme

Based on the ECC’s mutual authentication scheme by Karla and Sood as well as the capability of an adversary in the adversary model, we propose a lightweight block-chain based MFA scheme that integrates SSO and SAML in the cloud. The key agreement is further discussed in the subsequent subsection.

Deployment phase: The service provider controls the system components and smart objects before their deployment. For example, to register a $V_{h}$, the service provider implements the following processes.

*First: *$Vh_{i}$ check the $P_{u}K+h(R_{I}D_{Vh_{x}}∥P_{r}K∥P_{u}k)\cdot R_{u}k+V_{h}(R_{I}D_{h_{v}^{*}∥R_{u}K}∥|V_{hx}^{\prime }∥P_{u}k_{Vhx}∥T_{S}1)\cdot V_{h}x_{1}^{\prime }={\mathrm{Cert}}_{V_{h}x}^{+}$.*P*. If it matches, $Vh_{i}$ continues if the condition is satisfied. It rejects the request and cancels the authentication process.
*Second: *$Vh_{i}$ now retrieves $R_{I}d_{Vh_{j}}$ as $R_{I}d_{Vh_{j}}=$$R_{I}d_{Vh_{j}}^{*}⊕h\left(R_{V_{h}x}∥R_{I}D_{Vh_{x}}∥T_{S}1\right)$ and generates ${\mathrm{Cert}}_{V_{h}}^{\prime }={\mathrm{Cert}}_{V_{h}}⊕h\left(TID_{V_{h}x}∥s_{Vh_{x},V_{h}}∥TS_{2}\right)$ $TC_{V_{h}x}=B_{V_{h}x}⊕h\left(R_{I}D_{Vh_{x}}∥TS_{1}∥R_{V_{h}x}\right),C_{V_{h}x}=h\left(TC_{V_{h}x}\right.$ $\left.∥TS_{2}\right)⊕h\left(s_{Vh_{x},V_{h}}∥TS_{2}∥Sx_{V_{h}x}∥R_{I}d_{Vh_{x}}\right),X_{i}=$ $h\left(Sx_{V_{h}x}∥s_{Vh_{x}},V_{h}∥K_{1}^{\prime }\right)+\left(∥C_{V_{h}x}∥R_{V_{h}}∥{\mathrm{Cert}}_{V_{h}}∥\right.$ $\left.R_{I}d_{V_{h}}∥R_{I}d_{Vh_{x}}∥T_{S}1∥T_{S}2\right)$, where the current timestamp is $T_{S}2$, to send a key to establish a request $\mu =\langle TID_{V_{h}x},X_{i},{\mathrm{Cert}}_{V_{h}}^{\prime },K_{1}^{\prime },C_{V_{h}x},T_{S}1\;T_{S}2\rangle$ to $Vh_{x}$.

Moreover, the service provider also loads the shared secrets $S_{x}$ of the vehicles associated with the certificate to advance the embedded digital signature.

#### 5.2.1. MFA Key-Agreement Phases

The key agreement phases in this context are executed between the users (P and Q) in an IoT-based environment through an end-to-end communication, and this is achieved based on the following step, leveraging the embedded Probabilistic Polynomial-Time Algorithm (ePPTA).

- Step 1: Authentication Request. User P (IoT device) instantiates a communication link to the server, S, by sending the requisite identification parameters (DA).
- Step 2: Registration with ePPTA and Computation. Server generates $P_{i}$ and $R_{i}$, which acts as a private key based on the following ePPTA mechanism.
  – An embedded Probabilistic polynomial Time Algorithm is applied to the DA and a new Hash for every $P_{i}$
  – A common session key is generated by both parties by relying on $P_{i}+D_{s}+Hsh$
- Step 3: Authentication Phase. Server transmits to ID and it is able to get any record

#### 5.2.2. MFA Based on ePPTA

Based on the key agreement, we propose an integrated/embedded Probabilistic Polynomial-Time Algorithm (PPTA)-adding Digital Signature, $D_{s}$ and a hash, Hsh, for every $P_{i}$ generated by *S*. Based on this, a strong $P_{i}$ that an adversary may not be able to interrupt is presented as follows:

$$T_{i}=H\left(R_{i}⊕H\left(X\right)\right)\;A_{i}^{\prime }=A_{j}xG,T_{i},ID_{i}\;Ai=H(Ri⊕H\left(X\right)⊕Pi⊕CK^{\prime })\;CK=H(R_{i}\|X\|\mathrm{EXP}-\mathrm{Time}\|ID_{i})\;Server\;Stores\;A^{\prime }i=A_{j}xG,T_{i},ID_{i}\;Server\;generates\;P_{i}+D_{s}+Hsh\;x⟶S^{\prime }\;Private\_key,EXP-Time\;\;\;\;\;\;\;\;S⟶\;\mathrm{sends}\;CK^{\prime }\;to\;D_{i}$$

This implies that, during the authentication phase, where a mutual authentication between $D_{i}$ and cloud server is generated and both parties agree on a common session key (newly generated) based on $P_{i}+D_{s}+Hsh$, which means that, when the embedded device calculates values of A${}^{\prime }$ and then checks if $P_{4}^{\prime }=P_{4}$, it has to be generated using a unique hash digest has every time it is changed (integrated PPTA with a security parameter) as is shown in Figure 2. This further means that, in a blockchain environment, the generated Hsh will be three times stronger given that the probabilistic polynomial-time algorithm has to undergo another Hsh and this will be as follows:

Step 1: Signing Px using a Ds

$$Sender-privatekey,P_{x}\;is\;generated\;Server-generates\;P_{i}+D_{s}+Hsh\;Message-signed\;using\;P_{x}\;Sender\;public\;key,\;P_{k}-generated\;Message\;decrypted\;using\;\;\;\;\;\;\;\;\;\;\;Sender^{\prime }s\;P_{k}\to P_{i}+D_{s}+Hsh$$

**Figure 3:**
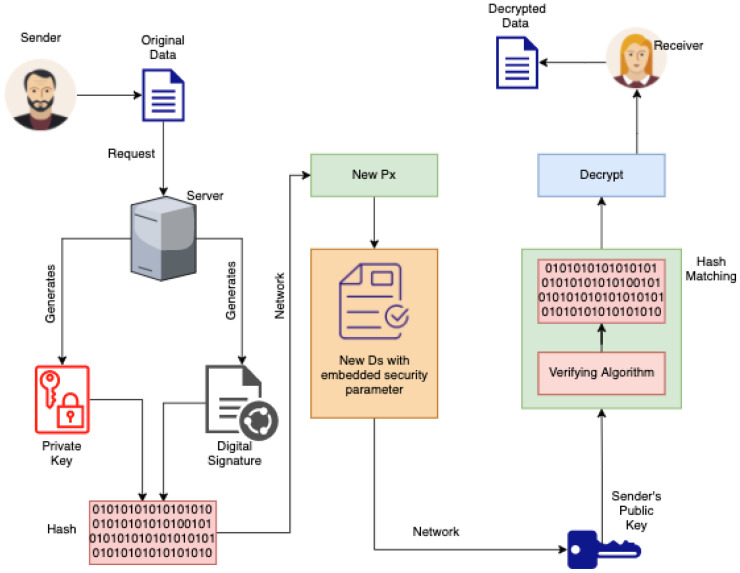
ePPTA with security parameter.

**Figure 4:**
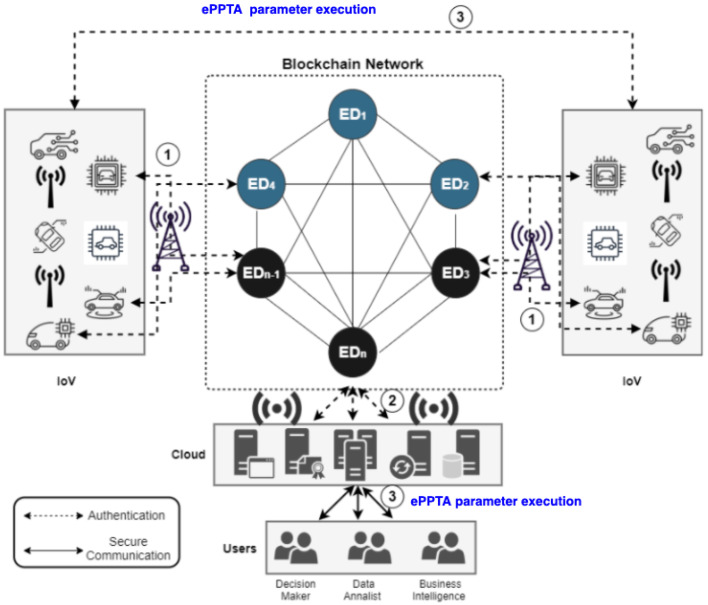
ePPTA with security parameter implementation in blockchain-based IoV.

Figure 3 which represents the ePPTA with a security parameter that hardens the MFA is implemented in Step 3 of the blockchain-based IoV model that was previously highlighted in Figure 2. Specifically, the ePPTA gives an assurance of access-control, confidentiality, and integrity. This also relies on the consensus made to the nodes in the blockchain network. Figure 4 shows the channel where ePPTA security parameter is implemented.

Step 2: Apply step 1 to SSO-SAML 

Through this step, the user can avoid further logins, and a directory of user details is maintained between the user and the Cloud Service Provider (CSP). The following requests are made in the SSO-SAML—$P_{i}+D_{s}+Hsh$ as follows: Supposing that a user wants to avoid multiple logins, it becomes imperative to maintain key details, which we posit as a Cloud Request, $C_{ij}-SAML$, and Cloud Application Request as $C_{App}-Rq$. The scheme requires the identification and authentication based on stored identities. For example, it allows matching bivariate polynomials f($C_{ij}-SAML$, $C_{App}-Rq$) over some degree p as is shown in Equation (1):

(1) $$f\left(C_{ij}-SAML,C_{App}-Rq\right)=\sum_{i,j=0}^{p}x_{i,j}{\left(C_{ij}-SAML\right)}^{i},{\left(C_{App}-Rq\right)}^{j}\left(x_{i,j}=x_{j,i}\right)$$

This ensures that every user’s identity can be requested based on the identity provider, $IDP_{Rq}$, which is mapped to the security parameter as follows:

(2) $$IDP_{Rq}⟼P_{i}+D_{s}+Hsh$$

For secure authentication, other relevant tasks accomplished in this step include Cloud Application Logging, $C_{App}-Log$, SAML Verification, $VRF_{SAML}$, and the user being able to access the cloud application, $USR_{Acc}-CApp$.

If there is a remote application, it can give the identity of the user based on the origin. In the context of this research paper, the origin may represent sub-domains used in the web or the IP addresses. The user is then able to be redirected to the IDP to request for authentication $Auth_{Rq}$. After this, the iDP can establish a logging connection over the browser section. An Authentication Response $Auth_{Rp}$ is built by the IDP which is represented by an XML-doc that consists of the user’s detail. These details are then transferred to the CSP through the $ACK_{sso}$ and $Rly_{Tgt}$. Eventually, the identity of the perceived cloud user can easily be established, and the CSP is able to transmit $CSP_{Trsmn}$. By employing this mechanism, the proposed approach can effectively prevent device/node hijacking as well as a spoofing attack within the communication channel.

The SSO service request and response occurs *n* and *m* number of times, respectively. This means that there may be distinctively *n* authentication modalities with *d* authenticating devices. Precisely, each authenticating modality possesses some characteristics c. We represent the authenticating modalities based on the characteristics as:

(3) $$n=\{n_{1},n_{2}\ldots n_{n}\}$$

and also with the modalities characteristics as is shown in Equation (4)

(4) $$n_{c}=\left\{c=1,2,\ldots n\right\}$$

The number of authenticating devices are represented as is shown in Equation (5)

(5) $$d=\{d_{1},d_{2}\ldots d_{n}\}$$

Therefore, the total authenticating modalities, features, and authenticating devices with their characteristics are represented based on Equation (6) given some degree *p* as follows:

(6) $$f\left(n_{c},d\right)=\sum_{i,j=0}^{p}x_{i,j}{\left(n_{c}\right)}^{i},{\left(d\right)}^{j}\left(x_{i,j}=x_{j,i}\right)$$

The process starts with a request from the service provider $SP_{Rq}$ to the user, which allows the user to register with the authentication server. This is then followed by the transmission of an SSO request $Trsmn_{SSORq}$ and an acknowledgment $ACK_{SSORq}$ to the identity provider and a request for key generation, $Keygen_{Rq}$ and $ACK_{keygen}$ to the SSO agent. After this request, the SSO agent can easily generate either a public or a private key and then the agent can be able to send the public key to the authenticating server. Finally, the authenticating server can generate the signature.

Step 3: Apply the New digital signature to the Blockchain 

We present a decentralized IoT smart city architecture that employs blockchain technologies that are centered on the multi-factor authentication approach mentioned in Step 1. The proposed architecture distributes the New Ds, over transactions as a $New_{Ds}$ * Ds + Proof-of-work(PoW) + Hash, which makes it infeasible to compute to any non-participating member. This mechanism can, therefore, foil the classical MITM, which SSO mechanisms are largely vulnerable to.

**Figure 5:**
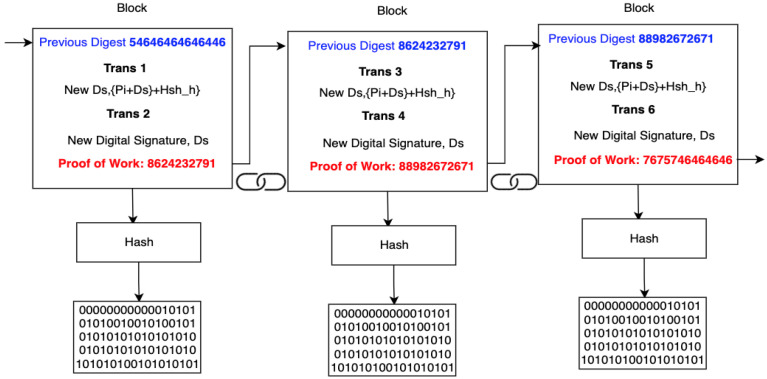
Figure depicting a Secure blockchain based on the current and new digital signature with a combined hash.

Every smart city can easily participate in normal transactions and communication can easily be effective over the distributed network. Our architecture integrates all transactions by incorporating a secure blockchain that has multi-factor authentication protocols that integrate SSO and SAML in the cloud and $New_{Ds}*Ds+Proof-of-work\left(PoW\right)+Hash$. Most importantly, each transaction is hardened using the sequence shown in Figure 5.

Each transaction $T=t_{1},t_{2},\ldots \ldots t_{n}$ on a given smart city blockchain is a validator that allows new members into the block to hold the new digital signature $New_{Ds}*Ds+Proof-of-work\left(PoW\right)+Hash$. This allows all the peers to validate the new peers using the most recent and longest Proof-of-Work. Unusual transactions that are not validated using $P_{i}+D_{s}+Hsh$ will be rejected. Peers can only be added to the blockchain network once a given transaction generates the $P_{x}$ and $P_{k}$ that are used during a normal transaction.

The PoW in the blockchain reduces the authentication and computation time needed from the scheme to the SAML SSO. It is worth noting that the proposed scheme can easily be applied to any blockchain system since it is secure in all means due to computational infeasibility of transactions because of immutable protocols. We also take note of the fact that the energy consumption by peers or attackers may be a point of interest in the blockchain. This study, therefore, prioritizes this as a major threat to the scheme. The new authentication scheme that is implemented in the blockchain is shown next:

$$T_{i}=H(R_{i}⊕H\left(X\right)\;A^{\prime }i=A_{j}xG,T_{i},ID_{i}\;Ai=H(Ri⊕H\left(X\right)⊕P_{i}+D_{s}+Hsh⊕CK^{\prime }\;CK=H(R_{i}\|X\|\mathrm{EXP}-\mathrm{Time}\|ID_{i}\;Server\;Stores\;A^{\prime }i=A_{j}xG,T_{i},ID_{i}\;ServergeneratesP_{i}+D_{s}+Hsh\;x\to S^{\prime }\;private-key,EXP-time\left(expiration\;of\;time\right)\;S⟶\mathrm{sends}\;CK^{\prime }\;to\;D_{i}\;Sender-private-key,P_{x}-generated\;Server\;generates\;P_{i}+D_{s}+Hsh\;Message-signed\;using\;P_{x}\;Sender-public\_key,P_{k}-generated\;Message-decrypted\;using\;Senders-P_{k},P_{i}+D_{s}+Hsh\;SSO-SAML+P_{i}+D_{s}+Hsh\;New-D_{s}\;Block:\;Trans_{n}+P_{i}+D_{s}+Hsh+PoW\to Hash$$

## 6. Discussion

As per the precise proposition that has been highlighted in this study, it is worth noting that the security techniques for an IoV are strengthened. The proposed mechanism of an embedded digital signature, which uses asymmetric encryption, aims to improve the PPTA from adversaries. Nevertheless, the proposed scheme follows an MFA technique that allows a user to authenticate several steps in the cloud while at the same time integrating with SAML-SSO. This approach is successful because there is a robust key generation procedure from the cloud server when an embedded device New Ds × Ds + Proof-of-work (PoW) + Hash attempts to connect to the server, S. This is because an embedded digital signature is applied in the immutable ledgers in blockchain transactions. Consequently, several security factors have been taken into consideration, given that it is computationally complex to change the functional requirement of any block within the blockchain during the exchange of transactions and ledgers. This is mainly because the peers in a blockchain will tend to trust the longest PoW that is generated from the blockchain. This implies that our approach adds a security layer to the PPTA, making it computationally infeasible during an attack, thereby creating a significant degree of trust, confidentiality, and integrity.

The proposed approach holds a direct data privacy impact on IoT applications such as smart cities. The realization of smart cities depends on individual data privacy and security to ensure realizing its vision and widespread its adoption among practitioners. However, such a vision faces challenges that include privacy preservation with high dimensional data, securing a network with a large attack surface, establishing trustworthy applications, properly utilizing artificial intelligence, and mitigating failures cascading through the intelligent network [44]. It is also essential to pay attention to the privacy solution impact on the system’s overall performance and employ state-of-the-art technologies like blockchain [45]. Further research directions utilizing our approach, hence, encourage further exploration of smart city deployment seeking privacy and performance.

A comparative analysis of the proposed approach with existing solutions is further given in Table 2. It can be observed that the proposed approach addresses key security objectives which were not considered in some earlier studies. Namely, we elaborate on data confidentiality and integrity. Further elaboration of these security objectives is discussed in the subsequent subsections.

### 6.1. Confidentiality

An adversary may want to intercept sensor data using various techniques, for example, through MiTM; however, the proposed scheme provides stronger approaches of an embedded digital signature that uses a private key, Px, and public key, Pk, to generate a new digital signature to compute the Proof-of-Work (PoW). Therefore, this implies that confidentiality is assured because any attempt by an adversary to eavesdrop on a communication would require a computationally complex attack path. Thus, attack during normal transactions in a decentralized smart city transaction can be said to be computationally infeasible. Notably, if an adversary tries to eavesdrop, conduct a brute force, or change the immutability of the blockchain, an adversary will need to compute quadrillions of computations to generate the blockchain hash because the embedded process has New Ds × Ds + Proof-of-work (PoW) + Hash.

### 6.2. Data Integrity

The possible attack path of an adversary is hampered by the proposed scheme in this context. This is because an adversary would typically attempt to alter the signed message through falsifying the contents. However, in this scheme, this is not feasible because the proposed authentication scheme employs a double computation $BlockTrans_{n}+P_{i}+D_{s}+Hsh+PoW\to Hash$ which makes transactions unmodifiable in a blockchain. Additionally, this also defeats the MiTM attacks or a mining attack in which the blockchain miners posing as adversaries may decide to control the cluster heads. This type of attack has been addressed in several existing studies, as highlighted in Table 2. This study can be added to the list of other studies whose security schemes provide a mitigation against this form of attack.

Table 2 shows the security attributes of closely related schemes and they have been used to show the evaluation of the proposed scheme. The attributes have been used to show a comparative security analysis between the schemes by [28,29,32,40,41]. The proposed scheme is based on IoV and applied in a blockchain environment and integrates SSO-SAML, while it resists MiTM and DoS, by enforcing confidentiality, integrity, and anonymity. In addition, the proposed scheme is precise for it has less cryptographic computations of the New Ds × Ds + Proof-of-work (PoW) + Hash to allow less energy usage during blockchain computations.

Furthermore, the approach provides a tamper-proof free approach for the sensor data from sensor nodes which are more vulnerable to attacks. One potentially added advantage of the proposed approach is the reduction in human activity. By leveraging the seamless characteristics of the SSO, and the security strength of MFA schemes based on block chain, the proposed approach presents a manageable approach to implement effective security in smart cities. Given that IoV based systems require a greater degree of automation and seamless communication, the proposed approach is suitable for the current high-speed 5G interconnected smart cities. Whilst the integration of blockchain presents a conceptual drift towards autonomous security in an IoV-centered platform such as smart cities, there are numerous potential adoptions of this integrated security. For instance, as observed in [46], the implementation of IoT-enabled platform cuts across numerous domains, ranging from smart health, smart education, and smart homes to smart offices. By extension, therefore, this proposed approach can be leveraged in any IoV-based platform for a secure seamless automation process. In terms of security, this proposed approach provides a relatively similar security strength to previous studies. However, the flexibility and ease-of-use of security have been overlooked. Usable security is fundamentally a component of security that has proven to aid technology adoption and enhanced security [47,48,49]. Thus, within the context of an IoV platform, a usable security would require an effective authentication process that provides a seamless and time-limited operations for connected vehicles.

On the other hand, the power consumption in Edge deployment architecture is one of the main concerns, limiting a full expansion of large-scale data analytics over IoT models [50,51]. It is expected that, alongside addressing the security and privacy concerns, advances in energy consumption will lead to the development of cross-devices Edge Intelligence applications and architectures [52]. This is especially required in mobile edge applications and sensor mesh networks (e.g., wearable sensors). However, the power consumption in the connected vehicle’s model is more resilient considering the vehicle’s power capacity, unlike the edge side unites [53]. In this regard, the main focus of this study is pushing the current research status a step further, realizing a secure Edge Intelligence paradigm. Hence, by investigating cutting-edge technologies (e.g., blockchain-enabled IoV [54]) and well-established identification and access control technologies, we aimed at a resident, scalable, and secure IoV system.

### 6.3. Distributed Attacks

Blockchain technology is becoming increasingly attractive, affecting the next generation of large-scale distributed systems, providing the required privacy. The blockchain theory relies on storing information securely within the blocks of the blockchain’s transactions. These decentralized consensus model transactions have the three main features: consistency, aliveness, and fault tolerance by nature [55]. However, in such an operational environment, distributed attacks are a leading concern [56]. Such an attack surface can return with transaction denial, as well as blockchain delay. Another attack dimension is punitive forking blockchain attacks where related transaction costs increased by peers in the blockchain, discouraging the production of non-renewable energy under certain circumstances [57]. The authors formalize the feather forking attack and we discuss how it can be applied in the smart grid context for the proposed purpose. They had further proposed a smart grid architecture addressing energy waste and production. In this context, we argue that combining the ePPT algorithm to the proposed blockchain-based identity and access control relieves such concern.

The proposed blockchain-powered approach enables different privacy-preserving models for IoT applications, such as data privacy, user privacy, location privacy, and privacy-preserving aggregation. Such a proposal aids in moving toward various advantageous features such as decentralization, anonymity, and audibility of the authentication process [58]. Hence, MFBC_eDS is a scalable and decentralized system with fast confirmation in the blockchain system. It uses a novel adaptive algorithm to integrate the Security Assertion Mark-up Language (SAML) to the Single Sign-On (SSO) capabilities. By combining these two strategies into an integrated consensus protocol, Blockchain smart contracts can be deployed as future work. This trend can capitalize on our proposed approach, strengthening against different threats, vulnerabilities, and attacks.

## 7. Conclusions

The integration of cloud computing and Vehicular Ad-hoc Networks (VANETs), namely, cloud-enabled IoV, has become a significant research area. This integration was proposed to accelerate the adoption of intelligent transportation systems. However, such a trend requires security mechanisms, ensuring data privacy, information integrity, and resource availability. In this paper, we explored the potential of a Blockchain-based Multi-Factor Authentication (MFA) model for the confidentiality and integrity of connected Internet-of-Vehicles (ioV). The proposed model integrates the Security Assertion Mark-up Language (SAML) to the Single Sign-On (SSO) capabilities for a connected ecosystem in the cloud. The evaluation reveals that the proposed model presents a reliable mechanism for enhancing the security of IoT-to-Cloud connected vehicles. In addition, this study presents the vision and need for robust access control in connected vehicle systems and fosters discussion on the identified future research agenda. We envision that this contribution will help achieve consensus among formal IoV access control models and real-world Cloud-Enabled IoV Platforms. As part of continuing work, parameters such as trust and malicious intention will be further explored to underscore the degree of reliability of the proposed solution. Device and user attribution within an IoV platform is another area of potential future work. Such future work involves developing several use-cases of malicious intention, where behavioral intentions can be modeled. The attribution process, on the other hand, can be used in a behavioral model. Taken together, therefore, the future work towards a reliable IoV authentication process would consider an extensive study of the uses cases that leverages behavioral model and attribution processes.

## Figures and Tables

**Table 1:** Formal BCMF_eDS authentication Model Definitions.

**Basic Sets and Functions** – $Vh_{i}$ is a finite set of Vehicles that is (i=1,2,⋯,n) and SP being the trusted service provider authority. – $P_{u}K$ and $P_{r}K$ are the Public and Private keys of each $Vh_{i}$. – $H_{i}$, $R_{ID}$, $S_{ID}$, and $T_{S}$ are hash function, Real Identification, Secret Identification, and a Time Stamp, respectively. – For any probabilistic polynomial time adversary, a probabilistic polynomial-time generates a $T_{S}$ for each $R_{ID}$ and $S_{ID}$ by adding a new $H_{i}$ for every $Vh_{i}$. – T is an upper bounded set of a subset *X* of some preordered set ($T_{K}$, ≤) is an element of $T_{K}$ which is greater than or equal to every element of X ∥∥, and the size of the input for the $T_{S}$ is $T_{S}\left(n\right)=O\left(n_{k}^{T}\right)$ for some positive constant k. – The selection algorithm sort based on m integers performs $Fm^{2}$ operations for some constant *F*. Time is a polynomial time algorithm and runs in $O(m^{2}$). – ePPTA: common session set when {ePPTA→ $H_{i}\}$. Formally, $H_{i}+R_{ID}+T_{S}+H_{i}$. – Each $Vh_{i}$ in the system maps $P_{r}K$, and *ID* in to a secret value. – ePPTA: $H_{i}\cup R_{ID}\cup S_{ID}\cup T_{S}\left\{Request\right\}\to T_{i}=H\left(P_{r}K⊕H\left(X\right)\right)$: $\left\{\begin{array}{c} A_{i}^{\prime }=A_{j}xG,T_{i},ID_{i}\\ A_{i}=H\left(P_{r}K⊕H\left(X\right)⊕P_{u}K⊕{\mathrm{CK}}^{\prime }\right) \end{array}\right.$
**Effective Authentication, MFA Based on ePPTA (Derived Functions)** – For each attribute att in ATT such that attType(att) = set: $CK=H\left(P_{r}K∥X∥\mathrm{EXP}\right.$-Time $\left.∥{ID}_{i}\right)$ Server Stores: $A_{i}^{\prime }=A_{j}\times G,T_{i},ID_{i}$ Server generates: $P_{u}K+S_{x}+Hsh$ $x⟶S^{\prime }P_{r}K,\mathrm{EXP}\_\mathrm{Time}\;∥∥\;S⟶$ sends $CK^{\prime }$ to $S_{i}$ ePPTA → $\left\{\begin{array}{cc} \left(\frac{1}{2}-\frac{1}{2^{n+1}}<\frac{1}{2}\right) & \mathrm{If}\;\mathrm{the}\;\mathrm{Algorithm}\;\mathrm{is}\;\mathrm{unsatisfiable}\\ \left(\frac{1}{2}-\frac{1}{2^{n+1}}\right)\cdot \left(1-\frac{1}{2^{n}}\right)+1\cdot \frac{1}{2^{n}}=\frac{1}{2}+\frac{1}{2^{2n+1}}>\frac{1}{2} & \mathrm{If}\;\mathrm{there}\;\mathrm{exists}\;a\;\mathrm{satisfying}\;\mathrm{assignment} \end{array}\right.$
**Authorization Functions and Decision Made** 1. $Vh_{i}$ confirms the received timestamp $T_{S}$ by checking if $\left|T_{S}-TS^{*}\right|\leq \Delta T_{S}$, where $T_{S}^{*}$ is the reception time of μ (the message). If the condition does not hold, $Vh_{i}$ stops further processing. Otherwise, $Vh_{i}$ fetches $\left(R_{I}D_{Vh_{x}},R_{Vh_{x}}\right)$ of the vehicle $Vh_{i}$ based on the received temporal identity $S_{I}D_{Vh_{x}}$ 2. $Vh_{i}$ checks that $P_{u}K+h\left(R_{I}D_{Vh_{x}}∥P_{r}K∥\right.\left.P_{u}k\right)\cdot R_{u}k+V_{h}\left(R_{I}D_{h_{v}^{*}}∥R_{u}K∥V_{h_{x}}^{\prime }∥P_{u}kV_{hx}∥\right.$ $\left.T_{S}1\right)\cdot V_{h}x_{1}^{\prime }={\mathrm{Cert}}_{V_{h}x}^{+}.P.$ If it matches, $Vh_{i}$ continues if the condition is satisfied. It rejects the request and cancels the authentication process. 3. $Vh_{i}$ now retrieves $R_{I}d_{Vh_{j}}$ as $R_{I}d_{Vh_{j}}=R_{I}d_{Vh_{j}}^{*}⊕h\left(R_{V_{hx}}∥R_{I}D_{Vh_{x}}∥T_{S}1\right)$ and generates ${\mathrm{Cert}}_{V_{h}}^{\prime }={\mathrm{Cert}}_{V_{h}}⊕h\left(TID_{V_{h}x}∥s_{Vh_{x},V_{h}}∥TS_{2}\right)$ $TC_{V_{h}x}=B_{V_{h}x}⊕h\left(R_{I}D_{Vh_{x}}∥TS_{1}∥R_{V_{h}x}\right),C_{V_{h}x}=h\left(TC_{V_{h}x}\right.$$\left.∥TS_{2}\right)⊕h\left(s_{Vh_{x},V_{h}}∥TS_{2}∥Sx_{V_{h}x}∥R_{I}d_{Vh_{x}}\right)$, $X_{i}=h\left(Sx_{V_{h}x}∥s_{Vh_{x}},V_{h}∥K_{1}^{\prime }∥C_{V_{h}x}∥R_{V_{h}}∥{\mathrm{Cert}}_{V_{h}}∥\right)$ $\left.R_{I}d_{V_{h}}∥R_{I}d_{Vh_{x}}∥T_{S}1∥T_{S}2\right)$, where the current timestamp is $T_{S}2$, to send a key to establish a request $\mu =\langle TID_{V_{h}x},X_{i},{\mathrm{Cert}}_{V_{h}}^{\prime },K_{1}^{\prime },C_{V_{h}x},T_{S}1\;T_{S}2\rangle \;\mathrm{to}\;Vh_{x}$ 4. $Vh_{i}$ creates $S_{x}{V_{h}x}^{\mathrm{new}}=S_{x}{V_{h}x}^{*}⊕h\left(S_{x}V_{h}x∥R_{I}D_{V_{h}x}∥R_{V_{h}x}∥T_{S}1\right)$ and updates $Sx_{V_{h}x}$ with $Sx_{Je}^{\mathrm{new}\;}$ for $V_{h}x$ in its secure IoV environment.

**Table 2:** Overview of a comparative summary of attributes.

Attributes	Proposed	Karla and Sood	Melki et al.	Wu et al.	Sharma	Xu et al.	Chin
MFA	✓	X	✓	X	✓	✓	X
SAML-SSO	✓	X	X	X	X	X	X
Confidentiality	✓	✓	✓	X	✓	✓	✓
Integrity	✓	X	✓	✓	✓	✓	✓
Anonymity	✓	✓	✓	✓	✓	✓	✓
IoV-centered	✓	✓	✓	✓	✓	✓	✓
Blockchain	✓	X	X	X	X	X	X

## Abbreviations

The following abbreviations are used in this manuscript:

ICT	Information and Communication Technology
IoV	Internet-of-Vehicle
MFBC_eDS	Multi-Factor Blockchain-based authentication model that uses an embedded Digital Signature
MFA	Multi-Factor Authentication
SAML	Security Assertion Mark-up Language
SSO	Single Sign-On
VC	Vehicular Cloud
IoT	Internet of Things
BYOD	Bring Your Own Device
OAuth	open authentication
SOPs	Standard Operating Procedures
ISPs	Internet Service Providers
SSL	Secure Socket Layer
MiTM	Man in the Middle
VANET	Vehicular Ad Hoc Networks
V2I	Vehicle to Infrastructure
PSO	Particle Swarm Optimization
WSN	Wireless Sensor Network
TA	Trusted Authority
RSU	Road Side Unit
ECC	Elliptic Curve Cryptography
$V_{h}$	Vehicle
V2V	Vehicle to Vehicle
*S*	Server
$D_{i}$	Embedded Device
$ID_{i}$	Identity
*N*	Randomized Number
$CK^{\prime }$	Authenticating Key
$P_{i}$	Key
$P_{u}K$	Public Key
$P_{r}K$	Private Key
$H_{i}$	Hash function
$R_{ID}$	Real Identification
$S_{ID}$	Secret Identification
$T_{S}$	Timestamp
$T_{S}\left(n\right)=O\left(n_{k}^{T}\right)$	size of the input for the $T_{S}$
ePPTA	Embedded Probabilistic Polynomial Time Algorithm
EXP−Time	Expiration Time
PoW	Proof of Work
$New_{Ds}$	New Digital Signature
Ds	Digital Signature
